# #childhoodobesity – A brief literature review of the role of social media in body image shaping and eating patterns among children and adolescents

**DOI:** 10.3389/fped.2022.993460

**Published:** 2022-08-29

**Authors:** Adriana Modrzejewska, Kamila Czepczor-Bernat, Justyna Modrzejewska, Agnieszka Roszkowska, Marcela Zembura, Paweł Matusik

**Affiliations:** ^1^Department of Psychology, School of Health Sciences in Katowice, Medical University of Silesia in Katowice, Katowice, Poland; ^2^Institute of Psychology, University of Wrocław, Wrocław, Poland; ^3^Institute of Pedagogy, University of Bielsko-Biala, Bielsko-Biala, Poland; ^4^Department of Pediatrics, Pediatric Obesity and Metabolic Bone Diseases, Faculty of Medical Sciences in Katowice, Medical University of Silesia, Katowice, Poland

**Keywords:** childhood obesity, social media, body image, eating patterns, children, adolescents

## Abstract

Children’s food preferences are closely related to their parents’ food preferences and knowledge of food is linked to what their parents share with them. Parents, however, are not the only people who model such behavior. Paradoxically, the ubiquitous technological development can also pose a huge threat. In developed countries, 94% of teenagers use social media platforms such as: Instagram, Snapchat, Facebook, or TikTok, and this also applies to children. It can therefore be argued that parents’ nutritional preferences and behavior are related to the same behaviors of children and there is an extensive literature on this subject. It is therefore important to check how other factors – new technology (and social media in particular) – can influence changes in this area. A literature search was conducted in the following databases: Google Scholar, PubMed, EBSCO in December 2021. After applying all the filters and verification of relevance in terms of the research on the topic of interest to us, 4 articles related to research on body image and social media and 4 articles related to research on food choices and social media among children and adolescents were obtained. The conducted analysis showed that various studies so far indicate that social media can have a very strong influence on the development of eating patterns and body image in children and adolescents, which in turn may be one of the risk factors for developing obesity when promoted behaviors are not associated with a healthy lifestyle. It is also worth pointing out that social media can be used as a resource in the prevention and treatment of obesity. A closer look at this topic seems to be particularly important due to the fact that, among adults, social media is not only a very important source of information about lifestyle, but also a source of social support when people attempting to lose weight. Therefore, by increasing preventive activity in social media and using modern solutions related to social media (including the use of hashtag signs), we can have a greater impact on the health awareness of children and adolescents around the world.

## Introduction

Childhood is the most important period in which eating habits are formed, which undoubtedly influences later health condition (1). The result of the transmission of incorrect eating patterns is obesity, other metabolic diseases and eating disorders (2–4). As is commonly known, these disorders are more and more often diagnosed in children and adolescents, and the recent situation related to COVID (e.g., due to the frequent change of lifestyle to a less healthy one) was conducive to the development of excessive body weight in this age group (e.g., (5–11).

The first educator in the field of eating behavior is the family (12). Research shows that children’s food preferences are closely related to their parents’ food preferences. Also, children’s knowledge of food is linked to what their parents share with them (13). It is well known that children learn to eat through their own experience as well as through the observation of others (14). It can therefore be argued that parents’ nutritional preferences and behavior are related to the same behaviors of children and there is an extensive literature on this subject (15–17). As the knowledge about eating behaviors is quite extensive, so it is important to check how new technology (and social media in particular) can influence changes in this area.

Parents, however, are not the only people who model such behavior (18). Paradoxically, the ubiquitous technological development, which is supposed to be a convenience, can also pose a huge threat (19). In developed countries, 94% of teenagers use social media platforms such as: Instagram, Snapchat, Facebook, or TikTok (20), and this also applies to children (21). Many studies around the world indicate that children and adolescents use social media for up to several hours a day, an example of such research is an Iranian study in which almost 80% of children and adolescents use social media 3-4 h or more a day (19). Using these media, we often come across information on the subject of obesity, which can very quickly spread around the world, including by using a hashtag (#) for them appropriately (22). For example, when analyzing the titular #childhoodobesity, on the one hand, we come across (good quality) educational materials that raise people’s awareness of the prevention and treatment of obesity. On the other hand, there are also numerous posts that can be strongly stigmatizing. The latter very often relate to two areas of functioning – body image and eating patterns (19). As shown by the research carried out so far, eating patterns and body image can play a very important role in developing obesity (e.g., (10, 23, 24), and one of the theoretical models explaining this is the Homeostatic Theory of Obesity (25). This model has also been empirically verified in studies conducted in a group of Polish children and adolescents (26). However, it is worth continuing research in this area to check how important social media in childhood are in shaping body image and eating patterns, especially given that (in general) children’s and adolescents’ use of social media has a significant impact on their body mass index (19).

Most children and adolescents publish their photos on the above-mentioned portals, so-called “selfies” (27). Considering the sociocultural model (28), teenagers internalize the ideals of appearance that are conveyed by the media and make comparisons with them (29, 30). According to the National Eating Disorders Association (31), body image is how an individual believes what they look like in the mirror, how they feel about their body, and how they feel in control about the body. This image can be positive or negative (31). The negative ones are an early indicator of an eating disorder. Regarding the shaping of one’s body image and exposure in social media, it is worth mentioning that social media promotes an unnaturally slim figure as desirable, often building a negative body image, and children and adolescents seem to be the most vulnerable group (31). This pattern often promotes stigma and strengthens the tendency of obese children to lose weight by means of maladaptive measures (e.g., very restrictive diets, use of laxatives, vomiting), which often lead to further weight gain in the long term (19, 32–34). Thus, as research shows, social media and peer groups functioning in it can, on the one hand, be an important source of knowledge and support in preventing obesity and promoting healthy growth (19, 35, 36). On the other hand, they can be a source of great discomfort, spreading myths about obesity and its treatment (19, 33). That is why it is so important to look carefully at how the content available on social media can influence the shaping of the body image and eating patterns of children and adolescents.

There is a strong international interest in research into eating behavior, the transmission of eating patterns through the family environment, and body image formation, but few studies in this context have analyzed the impact of social media (15–17). Importantly, not only selfies but also sharing pictures of meals with friends is also a popular phenomenon on social media (37). The reaction to such a photo is reacted on social media can also perpetuate and modify various eating and lifestyle behaviors. Therefore, this review of research aims to: (I) summarize the current research on the role of social media in the group of children and adolescents in (Ia) body image shaping, (Ib) shaping eating patterns, (II) indicating the essence of the problem and the direction of obesity preventive measures to public health institutions and other entities with a significant influence on the promotion of healthy eating behavior, mental health, and the proper use of social media, and (III) indicating further studies directions among obese children and adolescents in the context described above.

## Materials and methods

A literature search was conducted in the following databases: Google Scholar, PubMed, EBSCO from December 2021 to January 2022. The following keyword alone combinations were used: body image, body image and social media, food choices and social media, food choices, food choices and Facebook/TikTok/Instagram/Snapchat, body image and Facebook/TikTok/Instagram/

Snapchat. Filters in search engines were also used, such as: “Free full text,” ten-year articles, English and Polish, and the target research group “children and adolescents.”

**FIGURE 1:**
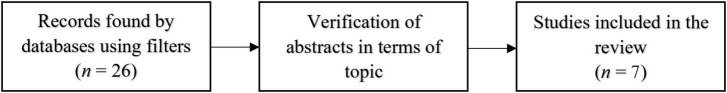
Flow chart of article selection process.

After applying all the filters, 26 results related to the subject of body image in social media and 8 results related to food choices in social media were obtained. Then, all the results were verified in terms of the relevance of the research on the topic of interest to us. As a result, 4 articles related to research on body image and social media among children and adolescents and 4 articles related to research on food choices and social media among children and adolescents were obtained. A block diagram of this process is shown in Figure 1. The “Supplementary Material” section provides more details about these studies (i.e., group description, variables and measures, results and detailed statistics for the measured variables; (Appendix Table 1)).

**TABLE 1:** Characteristic of the analyzed studies.

Authors (year)	Sample	Variables and measures	Descriptive statistics for variables and type of study
Steinsbekk et al. (41)	10 years old: *N* = 702 *M*_*age*_ = 10.51; *SD* = 0.17 12 years old: *N* = 668 *M*_*age*_ = 12.49; *SD* = 0.15 14 years old: *N* = 628 *M*_*age*_ = 14.33; *SD* = 0.59 Girls: 10 years old: 52.3% 12 years old: 51.9% 14 years old: 53.0% Boys: 10 years old: 47.7% 12 years old: 48.1% 14 years old: 47.0%	Social media use – report which social media platform use (e.g., Facebook, Instagram, Snapchat, Twitter) + characteristics of their use – self-oriented social media use: (a) the number of times per month they post something of their own social media sites, (b) how often they post photographs (never/rarely/weekly/daily) – other-oriented social media use: how often they commented on others” status updates and photographs, how often they “like”” others” statuses (6-point Likert scale) Appearance self-esteem 10-years old: *Self-Description Questionnaire* (SDQ-I; Marsh, (68) 12- and 14-years old: *Revised Self-Perception Profile for Adolescents* (SPPA-R; Harter (69); Wichstrøm (70)	Cross-secional study 10 years old: – *self-oriented social media use* (*M* = 10.33; *SD* = 26.15) – *other-oriented social media use* (*M* = 4.37; *SD* = 3.06) – *appearance self-esteem* – (*M* = 4.11; *SD* = 0.10) 12 years old: – *self-oriented social media use* (*M* = 17.95; *SD* = 30.60) – *other-oriented social media use* (*M* = 6.38; *SD* = 2.54) – *appearance self-esteem* – (*M* = 3.39; *SD* = 0.53) 14 years old: – *self-oriented social media use* (*M* = 15.02; *SD* = 30.19) – *other-oriented social media use* (*M* = 8.20; *SD* = 3.10) – *appearance self-esteem* – (*M* = 3.06; *SD* = 0.69)
de Vires et al. (29)	*N* = 440 *M*_*age*_ = 14.86; *SD* = 1.79 Girls: *N* = 205 Boys: *N* = 232 12 years: 12% 13 years: 18.2% 14 years: 11.8% 15 years: 13.4% 16 years: 22.6% 17 years: 18.7% 18 years: 3.2% 19 years: 0.2%	Body dissatisfaction *The Body Attitude Test* (BAT), (Probst et al. (71) Social media use *Multidimensional Scale of Facebook Use* (MSFU), (Frison and Eggermont (72) Relationship qualities *The Network of Relationship Questionnaire- Relationship Qualities Version* (NRI-RQV), (Buhrmester and Furman (73)	Cross-secional study *mean score of body dissatisfaction* (*M* = 2.81; *SD* = 1.43) – *mean score of social media use* (*M* = 3.92; *SD* = 1.41) – *a positive father-adolescent relationship measure (M* = 3.40; *SD* = 0.64) – *a positive mother-adolescent relationship measure (M* = 3.72; *SD* = 0.63)
Yang et al. (42)	*N* = 100 (female adolescents) *M*_*age*_ = 15.07; *SD* = 1.33 *M*_*BMI*_ = 19.05; *SD* = 3.45	Overall smartphone screen time *Smartphone Use* (questions: time on smartphone per day: sending and receiving e-mails, sending and receiving text messages, browsing websites, watching TV shows, taking photos, online shopping, listening to music) Daily frequency engaged in social networking sites *Media and Technology Usage and Attitudes Scale* (Rosen et al. (74) Cognitive internalization of thin ideals *the Sociocultural Attitudes Toward Comparison Scale-3* (Thompson et al. (75) Overall social comparison (online/offline) *The Physical Appearance Comparison Scale-Revised* (Schaefer and Thompson (76) Social appearance anxiety *Social Appearance Anxiety Scale* (Hart et al. (77) Body esteem *Body Esteem Scale for Adolescents and Adults* (Mendelson et al. (78) Internal control beliefs *Dieting Beliefs Scale* (Stotland and Zuroff (79)	Cross-secional study *smartphone screen time* (*M* = 4.10; *SD* = 1.41) – *website browsing* (*M* = 3.44; *SD* = 1.67) – *emailing* (*M* = 1.14; *SD* = 1.48) – *texting* (*M* = 3.34; *SD* = 1.60) – *listening to music* (*M* = 3.17; *SD* = 1.52) – *taking photos* (*M* = 1.70; *SD* = 1.34) – *taking videos* (*M* = 0.91; *SD* = 1.27) – *watching TV shows* (*M* = 3.21; *SD* = 1.58) – *online shopping* (*M* = 1.17; *SD* = 1.41) – *social media screen time* (*M* = 3.70; *SD* = 1.44) – *cognitive internalization* (*M* = 3.10; *SD* = 1.08) – *appearance comparison* (*M* = 5.74; *SD* = 2.20) – *appearance anxiety* (*M* = 3.37; *SD* = 0.98) – *body esteem* (*M* = 2.95; *SD* = 0.64) – *internal locus of control* (*M* = 3.33; *SD* = 0.34)
de Vires et al. (43)	*N* = 604 *M*_*age*_ = 14.7; *SD* = 1.7 *M*_*BMI*_ = 20.04; *SD* = 3.54 BMI: BMI under 30: 98.9% BMI under 25: 91.4% BMI under 20: 52.3% BMI under 18: 30%	Frequency of social network site use *Social Network Site Use -* questions about how often did you visit… in the past 6 months? Peer appearance-related feedback *Peer Appearance-Related Feedback -* questions about how often their friends (1) give them tips how to get a more beautiful body (2) criticize their appearance or clothes (3) give them tips how to look sexy (4) tell them it is important to look good Body dissatisfaction *The Body Areas Satisfaction Scale* (Cash (80) *The Multidimensional Body-Self Relations Questionnaire* (Cash (80)	Cross-secional study – *frequency of social network site use* (*M* = 2.4; *SD* = 1.5 – at time 1; *M* = 2.6; *SD* = 1.4 – at time 2) – *peer appearance-related feedback* (*M* = 0.53; *SD* = 0.57 -at time 1; *M* = 0.59; *SD* = 0.60 - at time 2) – *body dissatisfaction* (*M* = 1.46; *SD* = 0.65 - at time 1; *M* = 1.54; *SD* = 0.65 - at time 2)
Coates et al. (44)	*N* = 24 (six focus groups with children aged 10-11 years) One focus group: (*N* = 4)	Children”s understanding and attitudes about marketing *YouTube Video Featuring Influencer Marketing* (video “Nutella Breakfast Party””) *Photographs of Influencer Marketing Techniques* *Interview Guide*	Qualiative research	
Coates et al. (46)	YouTube videos (the authors two influencers - female aged 29, male – aged 24; both with a healthy weight).	*YouTube videos uploaded by two influencers*	Qualiative research Analysis of YouTube video blogs of influencers popular with children and determination of the extent and nature of food and beverage cues featured.	
Bragg et al. (49)	*N* = 832 *M*_*age*_ = 14.73; *SD* = 1.67 Male: *N* = 426 Female: *N* = 406	Instagram vs non-Instagram ads *Photo presentation and question:* How much do you like this image? *Photo presentation and question:* How artistic is this image? *Photo presentation and question:* How trendy is this image? *Photo presentation and question:* How delicious do you think this product is? *Photo presentation and question:* How likely are you to purchase this product in the next 4 weeks?	Cross-secional study	
			Instagram advertisement	Traditional advertisement
			Unlabeled advertisement condition/labeled advertisement condition *- How much do you like this image? (M* = 68.56; *SD* = 0.93/*M* = 67.43; *SD* = 1.04) *- How artistic is this image* (*M* = 68.58; *SD* = 0.93/*M* = 66.80; *SD* = 1.07) *- How trendy is this image* (*M* = 69.60; *SD* = 0.90/*M* = 68.16; *SD* = 1.02) *- How delicious do you think this product is?* (*M* = 66.80; *SD* = 0.94/*M* = 66.62; *SD* = 1.02) - *How likely are you to purchase this product in the next 4 weeks?* (*M* = 56.25; *SD* = 1.04/*M* = 56.03; *SD* = 1.38)	Unlabeled advertisement condition/labeled advertisement condition - *How much do you like this image?(M* = 65.72; *SD* = 0.93/*M* = 67.11; *SD* = 1.04) *- How artistic is this image* (*M* = 66.86; *SD* = 0.93/*M* = 66.84; *SD* = 1.07) *- How trendy is this image* (*M* = 66.28; *SD* = 0.90/*M* = 66.92; *SD* = 1.02) *- How delicious do you think this product is?* (*M* = 65.66; *SD* = 0.94/*M* = 67.51; *SD* = 1.02) - *How likely are you to purchase this product in the next 4 weeks?* (*M* = 55.27; *SD* = 1.23/*M* = 56.30; *SD* = 1.38)

## Results

### Body image and social media

Self-body perceptions, especially among girls in recent decades, have become a cause of global adolescent self-esteem (38). The basis of adolescent self-presentation is increasingly based on photos and videos on social media (39, 40). Social media can create appearance standards that are difficult to achieve (39), especially by adolescents and children. This situation can lead to lower self-esteem and emotional disturbances (41).

Previous research, for example, conducted in Norway on a group of 1998 respondents aged 10-14 (boys and girls), looked at which social media platforms they use, how often they post something on their account per month and how often they post photos of themselves and how often they comment on other people’s statuses and photos (41). Results indicate, among other things, that other-oriented social media use lowered self-reported appearance among respondents aged 10-12 and 12-14, while self-oriented use had no effect on this (41).

Another Dutch study, involved 440 teenagers of both genders aged 12 to 19 (29). The study was aimed at, *inter alia*, indicating whether the use of social media is a significant predictor of body dissatisfaction. The results showed that teens who reported more use of social media also reported higher levels of body dissatisfaction (29).

Interesting research in this area was also carried out in Singapore, where 100 female teenagers aged 13 to 18 were recruited from various local communities, such as the Chinese, Malayas, and Indians (42). Total smartphones use time, social media use, cognitive internalization, anxiety about social appearance, respect for the body, and position of weight control were assessed. The results suggest that only excessive use of social media, according to the authors, more than 3 h a day results in lower body evaluation results. Interestingly, the authors also explored the issue of engaging in online and offline appearance comparisons. They found that while social media escalates unhealthy cognitive patterns, it also does so outside of the time spent in these media, harming teenagers’ own body assessment, including a girl study (42).

A very different report is indicated by researchers from Denmark, who focused on the effects of social networking on body image among 604 adolescents (male and female) aged 11 to 18 (43). The study was related to the frequency of use of social media and information about the appearance that teenagers obtained from their peers and its impact on their body image. The results show that the more teens used social networking sites, the more often they received feedback about their appearance. Interestingly, the feedback received did not predict body dissatisfaction (43). This finding contradicts most studies about the association of social media with negative body image.

### Eating patterns and social media

Visual representations of food and beverage products in traditional communications and digital marketing primarily involve products high in fat, sugar and salt (44). YouTube is also very popular with children aged 5 to 15 (45), and the information contained in the content viewed influences their eating behavior (44). Research shows that exposure to food-related information contained in social media content, known as influencers, directly, immediately influences the choice and consumption of promoted foods by 9–11-year-old children (46, 47).

An equally interesting study on the marketing impact of a product promoted via the YouTube platform conducted in qualitative terms is a British study on a group of children (boys and girls) aged 10-11 years (48). The children watched a marketing video promoting the sweet product and were informed that the purpose of the study was to gather their views on YouTubers advertising food and drinks. The results of this study indicated that youtubers are a source of entertainment, information, social acceptance, and experiences for children, moreover, the products promoted by youtubers were desired by children (48).

The same authors also analyzed the channels of Youtubers popular among children to determine the scope and nature of the recommendations of food products and drinks (44). They also examined the proportion of “healthy” and “unhealthy” referrals. As it turned out, each of the commands had at least one food or drink tip, more often they were unhealthy than healthy. As many as 92.6% of the analyzed videos contained food and drink tips, which corresponds to 29.9 tips per hour (44).

A study by American researchers on a sample of 884 male and female adolescents aged 13-17 indicated that food ads posted on Instagram were very attractive to the respondents compared to traditional food ads (49). Interestingly, the Instagram symbol itself caused much more interest in the promoted product (49).

## Discussion

Various studies cited so far indicate that social media can have a very strong influence on the development of eating patterns and body image in children and adolescents, which in turn may be one of the risk factors for developing obesity when promoted behaviors are not associated with a healthy lifestyle.

Originally, the sociocultural model proposed by Thompson et al. (28) focused on traditional media, e.g., television, magazines, and the traditional “face-to-face” perception of the other person. Today, teenagers derive their ideal-looking messages from social media. According to the above-mentioned sociocultural model of comparison, the internalization of ideal appearance communicated through social media results in body dissatisfaction (50). This is supported by the analysis of the research presented in this article. In most studies on the impact of social media on body image, the target respondents are teenagers and adults and their results show a negative relationship between social media and body dissatisfaction (e.g., 43, 50–52). Therefore, attention should be paid to the importance of the problem of social media in the context of incorrect body image. If the problem is large among adult users of social media platforms, the group of children and adolescents may be even more at risk (53). Unfortunately, there are few studies that can approximate to the magnitude of this problem among children, and thus allow for the design of prevention activities aimed at child caregivers that would help monitor online behavior and allow for the protection of children from negative self-perception.

Social media also contributes to the promotion of food products to users (54). Influencers, youtubers show specific food products, recommend their purchase, and they are not always healthy (55). These are sponsored advertisements paid for by large food concerns. Research to date provides sufficient evidence of the effectiveness of influencer marketing on consumption primarily among adolescents (56). What is important for such marketing activities is that about 98% of people from the “Z” generation, i.e., people born after 1995, have a smartphone, and moreover, half of teenagers spend 10 or more hours a day using the telephone (57). It is therefore a powerful tool to influence choices, including food preferences. The Norwegian Consumer Council in 2019 showed that about 20% of all influencer-related marketing activities were for food and drinks (58). So far, research on this subject is not sufficient, and as far as we are aware, such research has not been conducted on a Polish sample. The exposure of children and adolescents to the influence of people for whom the most important thing is to sell a not necessarily healthy product is underestimated. Influencers often choose the way they present themselves on the Internet (59), using products and brands for self-presentation rather than actual consumption (60). The cognitive development of younger children (12 years of age and younger) is still developing, and thus a critical understanding of the commercial world will not be the same as the critical thinking skills of adults (61), therefore the popularity of influencers and content that they post on their profiles is particularly attractive to young audiences. Given this information, it is important to ensure the protection and control of young people in the digital space, and it should also be crucial for preventive health. To effectively protect children and adolescents, an intervention in the use of social media must be developed, and to implement it, a better understanding of how the use of social networking sites affects body image and food choices should be developed. Taking these actions is also important due to such phenomena as “echo chamber” (this can be defined as personalizing the content used on the Internet and matching it to the profile of a specific user, which means that we only receive information on social media that has been determined by appropriate algorithms as consistent with our interests and views; (62) and “mukbang” (this can be defined as an online audiovisual broadcast through a video-streaming platforms such as TikTok or YouTube in which a host consumes different amounts and types of food and interacts with the audience using a multimodal communication; (63), which may have a significant impact on shaping the awareness, body image and eating patterns of children and adolescents.

Summarizing the current knowledge, in future studies related to childhood obesity we should focus on: (I) analysis of the impact on eating patterns and body image of content from TikTok/Instagram/Snapchat, (II) taking into account the interaction of parents with social media in shaping un(healthy) eating patterns and (positive and negative) body image in their children and adolescents, (III) taking into account the assessment of children’s mental health (e.g., depression, eating disorders), (IV) taking into account children under the age of 10, (V) research among Polish children and adolescents including the division into genders, (VI) doing more experimental research in this topic.

Finally, it is also worth pointing out that social media can be used as a resource in the prevention and treatment of obesity. A closer look at this topic seems to be particularly important due to the fact that, among adults, social media is not only a very important source of information about lifestyle, but also a source of social support when people attempting to lose weight (e.g., 64, 65). Interestingly, this research shows that this online support is even greater than that they receive from their family and friends in the non-virtual world (64). Therefore, it would be interesting to check whether we recognize a similar effect in children and adolescents. Moreover, as is commonly known, many materials available on social media are not prepared on the basis of reliable and credible sources of information (e.g., 22, 66, 67). However, by increasing preventive activity in social media and using modern solutions related to social media (including the use of hashtag signs), we can have a greater impact on the health awareness of children and adolescents around the world, including fighting myths about obesity and patients who have been subject to stigmatization. Moreover, it seems clear that the topic of social media and their relationship with body image and eating patterns should be obligatorily addressed by psychologists and nutritionists during obesity therapy, thanks to which we can correct patients’ attitudes in this regard and increase knowledge and raise awareness among their caregivers.

## Author contributions

AM, KC-B, and JM conceived the study and performed literature search. All authors were involved in writing the manuscript and had final approval of the submitted and published versions.
